# Supplementary material for: Ultrafast and accurate 16S rRNA microbial community analysis using Kraken 2
Source: Microbiome. 2020 Aug 28;8:124. doi: 10.1186/s40168-020-00900-2 (PMC7455996; doi:10.1186/s40168-020-00900-2)
Supplement: Supplementary file 2 — Additional file 1 This file contains all command lines used for testing Kraken 2, Bracken, and QIIME 2 and with building each of the 16S rRNA databases. Additionally, this file contains a short discussion of the multi-threading behavior of QIIME 2. [file 40168_2020_900_MOESM1_ESM.pdf]

Supplemental Information

Contents

Kraken 2 and Bracken Command Lines **1**

1. Database Download/Building . . . 1
2. Classification/Abundance Estimation Steps . . . 1

QIIME 2 Command Lines **2**

1. Import Sequence and Taxonomy Files . . . 2
2. Train Classifier . . . 3
3. Import Sample . . . 3
4. Classification and Exporting Steps . . . 3

QIIME 2 Results Affected by Thread Count **4**

Kraken 2 and Bracken Command Lines

1. Database Download/Building

The following command lines were executed for each of the 16S databases (`${DBTYPE} = greengenes, silva, rdp`), using multi-threading options of 1, 4, 8, and 16 threads. As read lengths tested in this experiment are all 250bp, we used option `-l 250` for building the Bracken files.

```
kraken2-build --db ${DBNAME} --special ${DBTYPE} --threads ${THREADS}
bracken-build -d ${DBNAME} -t ${THREADS} -l 250 -k 35
```

2. Classification/Abundance Estimation Steps

For each sample and each database combination, we executed the following two commands tested using 1, 4, 8, and 16 threads.

```
kraken2
    --db ${DBNAME}
    --threads ${THREADS}
    --report ${SAMPLE}.kreport2
    --paired ${SAMPLE}_R1.fa ${SAMPLE}_R2.fa > ${SAMPLE}.kraken2
```

```
bracken -d ${DBNAME} -r 250 -l G -i ${SAMPLE}.kreport2 -o ${SAMPLE}_g.bracken
```

QIIME 2 Command Lines

All QIIME 2 command lines were executed within a conda environment. Command lines are displayed using multiple lines for clarity.

1. Import Sequence and Taxonomy Files

QIIME 2 compatible sequences for Greengenes 13_8 were downloaded from `ftp://greengenes.microbio.me/greengenes_release/gg_13_5/gg_13_8_otus.tar.gz`. The following command lines then were used to convert the files into QIIME 2 compatible files.

```
qiime tools import
    --type 'FeatureData[Sequence]'
    --input-path gg_13_8_otus_99.fasta
    --output-path gg_13_8_otus_16S.qza
qiime tools import --type 'FeatureData[Taxonomy]'
    --input-format HeaderlessTSVTaxonomyFormat
    --input-path 99_otu_taxonomy.txt
    --output-path gg_taxonomy.qza
```

QIIME 2 compatible sequences for SILVA 132 were downloaded from `https://www.arb-silva.de/download/archive/qiime/SILVA_132_release.zip`

```
qiime tools import
    --type 'FeatureData[Sequence]'
    --input-path silva132_99.fna
    --output-path 99_otus_silva.qza
qiime tools import
    --type 'FeatureData[Taxonomy]'
    --input-format HeaderlessTSVTaxonomyFormat
    --input-path 7_level_taxonomy.txt
    --output-path silva_taxonomy.qza
```

2. Train Classifier

```
qiime feature-classifier fit-classifier-naive-bayes
    --i-reference-reads gg_13_8_otus_16S.qza
    --i-reference-taxonomy gg_taxonomy.qza
    --o-classifier classifier_gg13_8.qza

qiime feature-classifier fit-classifier-naive-bayes
    --i-reference-reads 99_otus_silva.qza
    --i-reference-taxonomy silva_taxonomy.qza
    --o-classifier classifier_silva132.qza
```

3. Import Sample

QIIME 2 requires that all sample files first be converted into QIIME-compatible .qza files and that all sequences be dereplicated, generating a single sample file.

```
qiime tools import --type 'SampleData[Sequences]'
    --input-path combined_seqs_${SAMPLE}.fna
    --output-path  combined_seqs_${SAMPLE}.qza
qiime vsearch dereplicate-sequences
    --i-sequences combined_seqs_${SAMPLE}.qza
    --o-dereplicated-table table-${SAMPLE}.qza
    --o-dereplicated-sequences rep_seqs_${SAMPLE}.qza
```

4. Classification and Exporting Steps

The following command lines classifies the sample using the given trained classifier. This example uses the 16S Greengenes classifier. Additionally, this step allows for multi-threading. Therefore, we executed this command using 8 and 16 threads.

```
qiime feature-classifier classify-sklearn
    --i-classifier classifier_gg13_8.qza
    -i-reads rep_seqs_${SAMPLE}.qza
    --o-classification ${SAMPLE}_classsified_gg.qza
    --verbose
```

```
        --p-n-jobs ${THREADS}
```

Following classification, the QIIME file must be exported using the following steps.

```
    qiime tools export
        --input-path qiime_data/table-${SAMPLE}.qza
        --output-path ${SAMPLE}/
    qiime tools export
        --input-path qiime_classified/${SAMPLE}_classified_silva.qza
        --output-path ${SAMPLE}/
```

Following these two steps, we changed the first line of taxonomy.tsv to "#OTUID taxonomy confidence" separated by tabs

```
    biom add-metadata
        -i ${SAMPLE}/feature-table.biom
        -o ${SAMPLE}/${SAMPLE}-table-taxonomy.biom
        --observation-metadata-fp ${SAMPLE}/taxonomy.tsv
        --sc-separated taxonomy
    biom convert
        -i ${SAMPLE}/${SAMPLE}-table-taxonomy.biom
        -o ${SAMPLE}/${SAMPLE}-table-taxonomy.tsv
        --to-tsv
        --header-key taxonomy
```

QIIME 2 Results Affected by Thread Count

During our experiments, we discovered that changing the multi-threading options of QIIME 2's classification step affected the final results. Therefore, we tested the QIIME 2 16S Greengenes classifier using 1, 2, 4, 6, 8, 12, 16, 20, 24, 28, 30, and 32 threads and the human-gut microbiome sample. The results indicated that all results using 6-32 threads were identical, but different from the results using 1-4 threads. Additionally, we then were unable to replicate these results when executing the QIIME classifier commands at a different time.

Specifically, we calculated the MAPE error for the QIIME Greengenes classifier classifying the human gut

microbiome samples. The MAPE calculated from the initial test using 8 threads was 0.459. However, when we ran the classifier consecutively for 1-32 threads in our later test, 1-4 threads yielded results with a MAPE of 0.505 while results from runs with 6-32 threads yielded results with a MAPE of 0.421. We are unaware of what may cause the different results.
